# Experiences of assessing mental capacity in England and Wales: A large-scale survey of professionals

**DOI:** 10.12688/wellcomeopenres.16823.1

**Published:** 2021-06-10

**Authors:** Kevin Ariyo, Andrew McWilliams, Anthony S. David, Gareth S. Owen

**Affiliations:** 1 Mental Health, Ethics and Law Research Group, Department of Psychological Medicine, Institute of Psychiatry, Psychology and Neuroscience, King's College London, London, SE5 8AF, UK; 2 Metacognition Group, Wellcome Centre for Human Neuroimaging, University College London, London, WC1N 3BG, UK; 3 Experimental Psychology, University College London, 26 Bedford Way, London, WC1H 0AL, UK; 4 UCL Institute of Mental Health, Department of Psychiatry, University College London, London, W1T 7NF, UK

**Keywords:** mental capacity, capacity assessment, medical ethics, disability law, mental health law

## Abstract

**Background: **The Mental Capacity Act (2005) of England and Wales described in statute a test to determine whether a person lacked the “mental capacity” to make a particular decision. No large-scale survey has explored experiences of capacity assessment across professional groups.

**Methods: **We administered an opportunistic self-report questionnaire survey of professionals who undertake capacity assessments in England and Wales (n= 611). Topics of interest included; how often and where capacity assessment took place, self-ratings of competency and challenges experienced in assessment, use of psychological testing and concerns about undue influence. We analysed the quantitative responses using a mixed-methods approach using regression methods for the quantitative ratings and a thematic analysis for qualitative data.

**Results: **Our sample included 307/611 (50.2%), social workers, 89/611 (14.6%) psychiatrists, 62/611 (10.1%) nurses, 46/611 (7.5%) clinical psychologists, 30/611 (4.9%) doctors from other medical specialties, 12/611 (2.0%) speech and language therapists and 8/611 (1.3%) solicitors. 53% of these professionals undertook more than 25 capacity assessments per year, with psychiatrists, social workers and nurses undertaking them the most frequently. Most professionals reported high self-ratings of confidence in their assessment skills, although non-psychiatrist doctors rated themselves significantly lower than other groups (p< .005). Most professionals (77.1%) were at least moderately concerned about undue influence, with people with dementia and learning disabilities and older adults considered to be the most at risk. Qualitative themes for challenges in capacity assessment included inter-disciplinary working, complicated presentations and relational issues such as interpersonal influence. Requests for support mainly focused on practical issues.

**Conclusions:** Most professionals feel confident in their ability to assess capacity but note substantial challenges around practical and relational issues. Undue influence is a particularly common concern amongst professionals when working with service users with dementia and learning disabilities which public services and policy makers need to be mindful of.

## Introduction

The concept of mental capacity (or decision-making capacity) is fundamental to safeguarding the ethical principles of autonomy and informed consent. In England and Wales, the Mental Capacity Act 2005 (MCA 2005) and associated case law provide a legal definition of a test to determine whether a person aged 16 or over has the mental capacity to make a particular decision, and a framework to authorise an action to be taken in the best interests of a person who lacks capacity to take to a specific decision. Enshrined in the MCA 2005 are principles aiming to ensure that adults are supported to make informed decisions for themselves when they have the capacity to do so, and to have their views held at the core of the decision-making process even if they do not.

While the MCA 2005 has been heralded for its legal clarity, there are several ongoing debates over its implementation. Anyone can conduct or be subject to a capacity assessment at any time ( Brown *et al.*, 2015), so the challenges can vary considerably based on the person’s needs and impairment, as well as the professional’s skills and resources. To ensure high practice standards across the board, it is important to consider the full range of contexts in which these assessments can be applied. The recent National Institute for Care Excellence (NICE) guideline on decision making capacity outlined several research recommendations for health professionals who assess capacity ( National Institute for Health and Care Excellence, 2018). Their key priorities included outlining training and support needs, the components of a capacity assessment and the potential use of tools to facilitate the assessment process. These should help to ensure the provision of high quality, legislation-compliant assessments ( Jayes *et al.*, 2020a; Jayes *et al.*, 2020b) and to better engage with the most difficult aspects of assessing capacity, such as the ‘use or weigh’ criterion within the functional test ( Keene *et al.*, 2019).

Since the implementation of the MCA 2005 in 2007, only a small number of studies have explored the experiences of the professionals who carry out capacity assessments. These studies have each surveyed just one professional group (though some have included multiple sub-specialties of medicine), using opportunistic sampling, with fewer than 100 participants. Their findings have included: low confidence amongst doctors who assess capacity ( Penn *et al.*, 2020), concerns around under-utilisation of speech and language therapists ( McCormick *et al.*, 2017) and difficulties assessing brain injury patients with fluctuating capacity ( Norman *et al.*, 2018). These studies suggest that experiences of capacity assessment may be context-specific, which could be explored within a larger and more varied sample.

Furthermore, to our knowledge, this literature has not considered professional views about how third-party social influences affect a person’s mental capacity during assessments. For example, undue influence may result in a person being unable to exercise their capacity ( Mandelstam, 2013). These concerns have been raised on several occasions within the contested capacity case law (A Local Authority v Mrs A and Mr A [2010] EWHC 1549; Brent LBC & Ors v Risk Management Partners Ltd [2011] UKSC 7; PC v City of York Council [2013] EWCA Civ 478, [2013] MHLO 61), but it is currently unknown to what extent they are reflected in everyday capacity assessments. A more comprehensive understanding of these issues will be key to ensuring effective implementation of the MCA 2005.

The main objectives of our study were to describe and compare the experiences of professionals who assess capacity in England and Wales. We sought to include professionals with a range of experience and responsibilities, across the health care, social care and legal disciplines. We were particularly interested in four questions:

1. When, where and how often do professionals assess capacity?

2. How effective do professionals feel they are at assessing mental capacity?

3. How do professionals consider undue influence when assessing mental capacity?

4. What are the challenges experienced when conducting any structured assessments?

## Methods

### Design

We developed a questionnaire for a cross-sectional survey (see extended data) ( Ariyo *et al.*, 2021) to explore the experiences of professionals assessing mental capacity. It was developed using insights from the literature and from the clinical experience of the research group. To maximise engagement from professionals, the survey was short and anonymous. It included a mixture of fixed-choice, Likert-scale and free text questions.

We then piloted the questionnaire within the research team, incorporating revisions where warranted.

### Data collection

The web-based version of the survey was delivered via the academic survey provider onlinesurveys.ac.uk and was available from 07/02/2019 to 07/01/2020, in order to allow sufficient time to collect responses from a large sample and across different professions. Potential respondents were contacted using email and social networking sites, via professional networks and professionals with high profile social media presence. Hard paper copies of the same questionnaire were circulated at two academic conferences in London (in neuropsychiatry and in psychology). Partially completed questionnaires were included.

### Data analysis

***Quantitative analysis*.** Likert scale, yes/no responses and questions with numeric answers were analysed quantitatively, and K.A. performed the analyses using SPSS (version 27.0). We calculated descriptive statistics to determine means, standard deviations, normality of distributions (Shapiro-Wilk test and boxplots), homogeneity of variances (Levene’s test) and missing data within the numerical responses. We considered 5% missingness as a reasonable cause for concern and 10% as significant cause for concern ( Bennett, 2001; Schafer, 1999).

Following this, we used a series of one-way independent analyses of variance (ANOVAs) to explore differences between professionals to the following questions: how often they assessed mental capacity (Q4), how competent they felt in doing so (Q7), and how often they were concerned about undue influence (Q11). These were scored between one and five, with higher scores indicating higher values. As Q4 was originally scored on a four-point scale, we transformed each data point into a five-point scale for reporting purposes only, to ensure consistency.

The main effect of group differences is reported using eta squared (n ^2^), which is the most common effect size for ANOVA ( Cohen, 1973). We also calculated 95% confidence intervals using the bias-corrected and accelerated (BCa) bootstrap method, as the assumption of univariate normality was violated. Following a significant result, we conducted a Tukey’s post-hoc analysis to explore pairwise comparisons between professional groups (or the Games-Howell statistic when homogeneity of variances was violated).

***Qualitative analysis*.** Free text responses to one question, concerning the challenges experienced when assessing mental capacity, were analysed using a procedure based on the six steps of thematic analysis described by ( Braun & Clarke, 2006), as this method has a track record of use in healthcare and social science research ( Braun & Clarke, 2014).

The thematic analysis used a bottom-up, inductive, and experiential viewpoint ( Clarke & Braun, 2013), comprising an iterative process of coding, derivation of thematic structure and comparison. Two researchers undertook the analysis (KA and AMcW). 

We independently immersed ourselves in the responses, re-reading them while noting initial striking impressions, before each researcher independently applied to the responses codes which had emerged. Individual responses could be given more than one code, partial segments of responses could be coded, and every part of each response was placed within a code. We then compared the codes we had derived and, by referring back to the raw responses, agreed on a set of 51 codes. We then independently re-coded the responses and derived a prototype thematic structure. We continued an iterative process of coding and creating themes and subthemes until consensus was reached on both the coding and the thematic structure. We then chose the final names for the themes and subthemes they contained.

Although our survey generated free text in response to other questions, these generally were brief and somewhat factual responses, without sufficient elaboration to allow use of qualitative analysis. Instead, we outline the characteristics of the responses to portray the most salient features.

### Ethics statement

Our project was approved following a review by King’s College London’s College Research Ethics Committee (MRA-18/19-10500). An information sheet about the study describing how data would be used was provided to all participants. Participation in the survey was taken as implying consent.

## Results

### Sample characteristics

We received questionnaires from a total of 621 participants (573 online and 48 paper). Ten participants (nine online, one paper) were excluded for the following reasons: not a health, social care or legal professional (n=4), did not report their profession (n=3), only assessed capacity outside of England and Wales (n=2), only assessed capacity as part of a research study (n=1) ( Ariyo *et al.*, 2021).

Table 1 outlines all of the included participants by profession. The 30 non-psychiatrist doctors within the sample included general practitioners (n=8), obstetricians or gynaecologists (n=7), neurologists (n=6), geriatricians (n=4) and a neuro-rehabilitation specialist. Four doctors didn’t state their specialty. We also created a composite ‘other’ category for professional groups that were poorly represented in the sample. These included midwives (n=7), social care managers (n=7), best interest assessors (n=5), assistant psychologists (n=4), assistant social care workers/practitioners (n=4), physiotherapists (n=3), legal case workers (n=3) and other roles (n=8). We excluded these participants from the significance testing to avoid introducing additional heterogeneity.

**Table 1.** Descriptive characteristics of respondents by profession.

Profession	N (%) ^a^	% Expert witnesses ^a^	Most common decisions assessed ^a^	% ^a^
Clinical Psychologist	46	32.61%	Managing own finances	70.45%
			Accommodation choice	68.18%
Doctors (non-psychiatrist)	30	3.70%	Physical health treatment	82.76%
			Discharge from hospital against medical advice	44.83%
Nurse	62	4.92%	Accommodation choice	54.10%
			Physical health treatment	47.54%
Occupational therapist	16	0.00%	Accommodation choice	81.25%
			Other	43.75%
Psychiatrist	89	21.35%	Mental health treatment	82.02%
			Physical health treatment	75.28%
Social Worker	307	6.21%	Accommodation choice	79.80%
			Managing own finances	58.28%
Solicitor	8	n/a	Managing own finances	87.50%
			Accommodation choice	75.00%
SLTs	12	0.00%	Accommodation choice	58.33%
			Physical health treatment	58.33%
Other	41	2.50%	Accommodation choice	48.98%
			Physical health treatment	40.82%
Total	611	9.57%	Accommodation choice	65.63%
			Managing own finances	47.95%

^a^ Only totals include missing data; see Table 2. SLTs, speech and language therapists.

**Table 2.** An overview of missing data for the multiple-choice questions by each profession.

Q	Question	Missing (N)	Present (%)	N> 5%
3a	What is your profession?	0	100.00%	None
3b	If you indicated you are a medical doctor, what is your specialty?	0	100.00%	None
4	On approximately how many occasions do you formally assess capacity?	7	98.85%	6.8% of non- psychiatrist Doctors
5	Have you ever been an expert witness for capacity issues in court?	5	99.18%	10% of non-psychiatrist
6	Which decision(s) most commonly trigger a capacity assessment?	1	99.82%	None
7	How well do you feel you assess capacity?	5	99.18%	12.5% of Solicitors
11	How often are you concerned that the person’s wishes have been unduly influenced by other people?	1	99.84%	None

Of the 611 participants, 59 (9.57%) reported having been an expert witness for capacity issues in court, including 32.6% of the clinical psychologists and 21.4% of the psychiatrists in the sample. No occupational therapists or speech and language therapists reported having been expert witnesses for capacity issues.

Professionals most commonly reported having conducted capacity assessments in hospital/inpatient services (39.97%), care homes (33.88%), community/outpatient services (31.25%) or the person’s own home (27.47%). Only 1.48% of the sample most commonly assessed capacity in court settings. The two most common decisions that needed to be assessed were for accommodation (65.63%) and financial management (47.95%).

### Quantitative analyses

***How often do professionals assess capacity?*** The assumptions of univariate normality and homogeneity of variances were both violated (p< .05). A one-way Welch ANOVA with bootstrapping revealed that self-reported frequency in conducting capacity assessments significantly varied between professional groups, Welch’s F(7, 57.12)= 11.73, n ^2^= .140, p< .001, 95% BCa CI [.08, .18].

As shown in Table 3, there were significant differences between certain professional groups. Psychiatrists and nurses reported the highest frequency of assessments, whereas clinical psychologists, solicitors and non-psychiatrist doctors reported the lowest. 70.9% of psychiatrists and 68.9% of nurses reported having conducted at least 25 capacity assessments within the previous year.

**Table 3.** Respondent professions and characteristics of quantitative responses.

Profession	Assessment frequency score ^a^ mean (SD)	Significantly greater than ^d^	Self-rated competence score ^b^ mean (SD)	Significantly greater than ^d^	Undue influence score ^b^ mean (SD)	Significantly greater than > ^c^
Clinical Psychologists	2.36 (1.53)	None	3.78 (.81)	None	3.15 (.84)	Doctors, Psychiatrists
Doctors (non-psychiatrist)	3.19 (1.82)	None	3.45 (.95)	None	2.53 (.94)	None
Nurses	4.23 (1.28)	Psychologists, Doctors, OTs, Solicitors	4.11 (.73)	Psychologists, Doctors	2.89 (.79)	None
OTs	3.17 (1.75)	None	4.00 (.73)	Doctors	2.81 (.75)	None
Psychiatrists	4.46 (.99)	All except Nurses	4.07 (.68)	Doctors	2.74 (.82)	None
Social Workers	3.94 (1.40)	Psychologists, Doctors	4.04 (.67)	Doctors	2.94 (.79)	Doctors, Psychiatrists
Solicitors	2.83 (1.58)	None	3.71 (.76)	None	3.75 (.71)	All except SLTs
SLTs	3.67 (1.50)	Psychologists	4.33 (.65)	Psychologists, Doctors	3.33 (.99)	Doctors, Psychiatrists
Weighted mean ^e^	3.48		3.94		3.02	

^a^ Four-point scale transformed to five-point scale for reporting only. ^b^ Five-point scale (1= low, 5= high). ^c^ p< .05 according to Tukey’s test with bootstrapping as equal variances assumed. ^d^ p< .05 according to Games-Howell test with bootstrapping as equal variances not assumed. ^e^ Weighted mean (mean of each average score per professional group) to control for differences in sample sizes between professional groups. OT, occupational therapists; SLT, speech and language therapists; SD, standard deviation.

***How effectively do professionals feel they assess capacity?*** The assumptions of univariate normality and homogeneity of variances were both violated (p< .05). A one-way Welch ANOVA with bootstrapping revealed that self-reported competence in conducting assessments significantly varied between professional groups, Welch’s F(7, 54.09)= 2.66, n ^2^= 0.49, p= .02, 95% BCa CI [.01, .08].

As shown in Table 3, speech and language therapists, nurses, psychiatrists and social workers rated themselves highest in terms of assessing mental capacity, whereas psychologists rated themselves lowest (p< .05). 41.7% of speech and language therapists, 29.5% of nurses, 25.3% of psychiatrists and 23.6% of social workers rated themselves as being able to assess capacity ‘very well’. Only 19.57% of clinical psychologists rated themselves as such. The average score for self-rated competence was reasonably high overall (M= 3.94, SD= .74). Self-rated competence was also positively associated with the number of capacity assessments the professional undertook per year (p< .001).

***How often are professionals concerned of undue influence?*** The assumption of univariate normality was violated (p< .05) but equal variances were assumed (p> .05). A one-way ANOVA with bootstrapping revealed that professional groups differed in the extent to which they considered undue influence, F(7)= 3.85, p< .001, n ^2^= .46 95% BCa CI [.01, .07].

As shown in Table 3, solicitors reported suspecting undue influence the most often, and to a significantly greater extent than all other professions except for speech and language therapists (p< .05). Clinical psychologists also reported significantly greater suspicion than psychiatrists and non-psychiatrist doctors, who reported the least concern. The average level of suspicion was moderate overall (M= 3.02, SD= .83).

When asked to consider who was most vulnerable to undue influence, professionals’ responses were varied. Responses included older adults (35.3%), people with learning or intellectual disabilities (38.6%), dementia (17.8%) and severe mental illness (12.6%) as well as children and adolescents (8.8%), other groups (14%) or none specified (3.1%).

**Figure 1.**
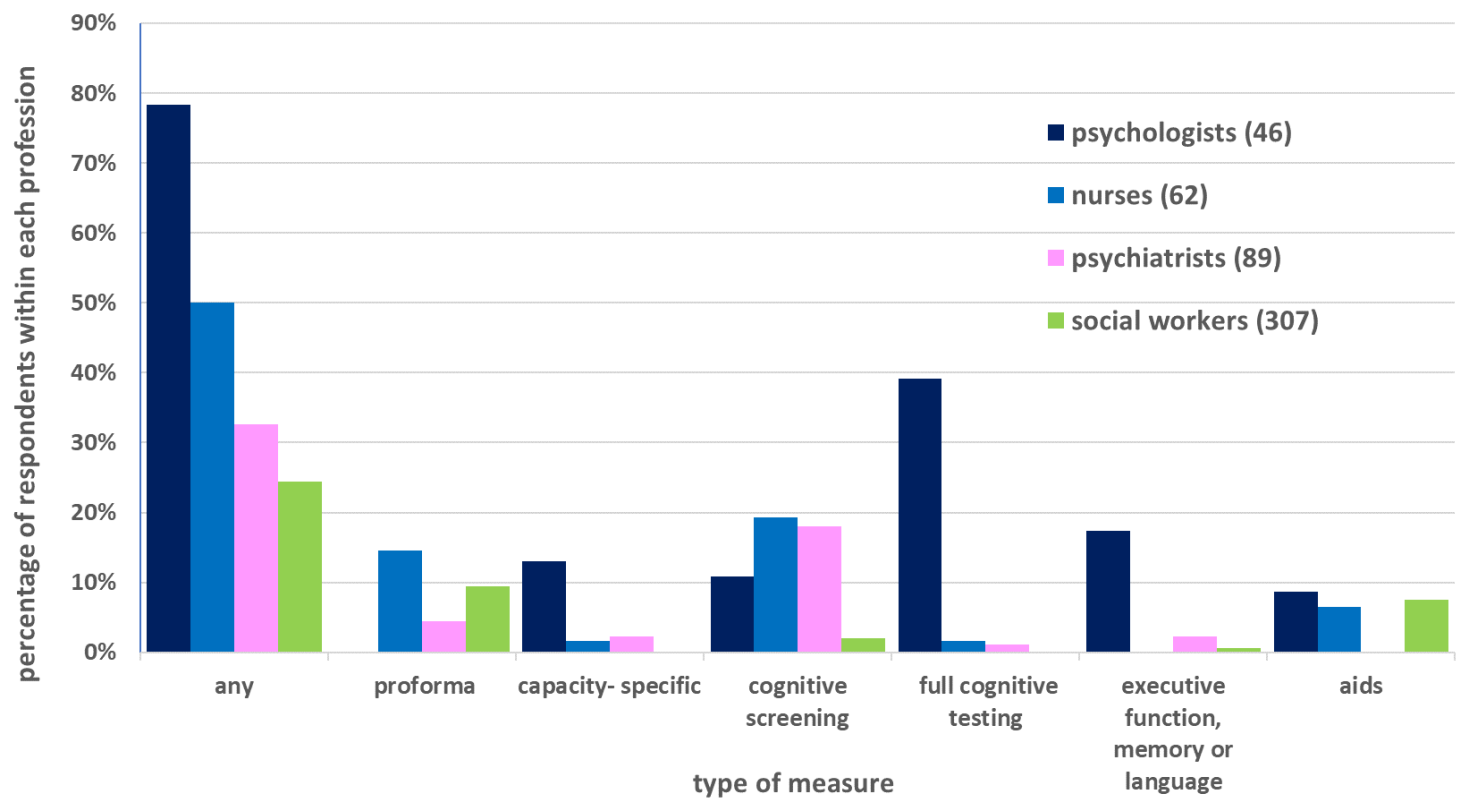
Use of psychometric measurement tools by the four largest professional groups.

***What are the challenges experienced when conducting any structured assessments?*** 210/611 (34.4%) of respondents indicated use of some kind of structured tool or psychological testing when assessing capacity. Figure 1 shows rates reported by the four largest professional groups, categorised by type of measure. The most common (61/611; 10.0%) type of tool used was a proforma of some kind to record capacity assessments, such as that made by the employer or third sector organisations, although no psychologist reported their use.

Screening tests for cognitive impairment were used by 46/611 (7.5%) respondents, including: Montreal Cognitive Assessment (MoCA) ( Nasreddine *et al.*, 2005) (21/611; 3.4%), a version of the Addenbrooke’s Cognitive Examination (ACE) ( Mioshi *et al.*, 2006) (19/611; 3.1%) or the mini-mental state examination (MMSE) ( Folstein *et al.*, 1975) (15/611; 2.5%). Full cognitive or “intelligence” testing was referred to in 21/611 (3.4%) responses, including: Wechsler Adult Intelligence Scale ( Wechsler, 1955) (9/611; 1.5%), followed by generic references to “IQ” (4/611; 0.65%). Quantification of executive functioning was described in 9/611 (1.5%), standalone tests of language (receptive or expressive) in 8/611 (1.3%), and of memory in 5/611 (0.82%).

Use of a tool designed with the specific purpose of assessing mental capacity was made by 9/611 (1.5%) respondents.

9/611 (1.5%) of respondents specified that structured tests must only be used to assess impairment of brain or mind (MCA 2005 s.2) and not to assess impairment of decisional function (MCA 2005 s.3). Sceptical or disparaging comments about the usefulness of structured and psychological tests for assessing mental capacity were made by 14/611 (2.4%).

### Qualitative analysis

Thematic analysis was performed separately on responses from two survey questions. The first (“What are the main challenges to capacity assessment?” n= 595) yielded nine themes relating to practices of capacity assessment. Analysis of the second question (“What would help you assess capacity better?” n=541) revealed responses which were generally much shorter, suggesting that respondents had attached less importance to this question. Responses generally repeated on those already given and generated no additional themes.

***What are the main challenges to capacity assessment?*** Table 4 shows the thematic structure of nine themes and the subthemes they contained, which we then describe, using illustrative quotations, each originating from different, distinct individuals. 

**Table 4.** The thematic structure of challenges to capacity assessments.

#	Themes	Sub-themes
#1	Practical issues	Time; settings; repeat assessments
#2	Interface with professionals	Responsibility and accountability; differing opinions; accessing specialist input; differing professional agendas; joint assessments as desirable
#3	Struggling to gather information	Working out information pertinent to a decision; refining interview questions; gathering collateral information
#4	Engagement and influence	Relationship building; engaging the person during assessment; family and carers; undue influence and coercion
#5	Supporting the person during the assessment	Providing communication support; anxiety and nerves
#6	Diagnosis as imperative	Knowing the diagnosis; specific named diagnoses; severe symptoms
#7	Navigating the functional test of capacity	Assessing functional abilities; assessing communication (without mentioning support); causative nexus; frontal lobe paradox; unwise decisions
#8	Complicated presentations and pressing decisions	Crisis presentations; urgent decisions; borderline capacity; fluctuating capacity; threshold issues
#9	Legal knowledge and opinions	Medico-legal practice; keeping up to date with case law; critiquing the law; guidance and training

*Theme 1: Practical issues*

A wide range of logistical issues with conducting assessments were reported consistently through the responses. Finding sufficient time for the assessment was a prime concern and shortages affected the possibility of developing a clear understanding of the decision before undertaking the assessment, obtaining background information on the person and triangulating that information when necessary:

*“Time to prepare to ensure I am completely familiar with P's circumstances to ensure the assessment is specifically tailored to P's current situation and their concrete options.”* Social worker

*“The main challenge is that organisational pressure means there is often not enough time. Time is often needed to make bespoke resources e.g. images to represent relevant information.”* Speech and Language Therapist

Time to get to know the person was also crucial for some respondents:

*“Having the time to get to know the person being assessed, how they think and how they communicate.”* Social worker

There was a lack of suitable private environments for encounters and inadequate documentation systems for assessment reports. Some participants stated it was impractical to make the multiple attendances they needed to complete an adequate assessment:

*“In my job only one visit is made to the client, generally this is sufficient but can cause issues when people are anxious about what is about to happen. I find clients perform better when they are relaxed about the process and depends on how they respond to me”* Nurse

*Theme 2: Interface with professionals*

Other professional groups could be seen as inaccessible, having different opinions and agendas when assessing capacity. Cutting across this theme were difficulties with determining who was best placed to undertake the assessment.

*“Other disciplines assuming it is my role to complete an assessment based on the grounds that I may know the client better than them; however as the decision being assessed is outside of my role, I would not be well placed to complete an assessment. This is often experienced as withholding by other professionals.”* Clinical psychologist

*“I would only carry out an informal capacity assessment. If I felt that a person was lacking in capacity I would then refer for a formal assessment, to which I am finding a reluctance from some health professionals dealing with P’s care in assisting with the assessment. This then leads to either a long wait time in finding another person to carry out the assessment, in that case can potentially leave a vulnerable person at risk without them being able to make decisions for themselves or not having an appointed person to make decisions on their behalf.”* Legal casework manager

If additional specialist support was required, either to give support during an assessment or to perform an additional one, this was not always available in a timely manner:

*“Organisational issues such as limited time and difficulties getting IMCA’s as they have huge caseloads in my area”* Social worker

Some professionals wanted the assessment to be undertaken by a team rather than an individual, with responsibility for the outcome dissipated across the group. Some participants expressed concerns that they were asked to provide second opinion assessments, but as they were not members of the team working with the person, it was difficult to understand fully the decision in question and to get to know the person sufficiently to allow a quality assessment:

*“…often asked to engage in these assessments but we may not know the individual best- other staff groups are likely to be in a more informed position to make a judgement but lack the confidence to do so”.* Clinical psychologist

There was also concern about where the responsibility for deciding issues of capacity lay when second opinion assessments has been requested:

*“Persuading my medical colleagues that the MCA COP[code of practice] is relevant to their practice and that they may in fact be the decision maker.”* Psychiatrist

*Theme 3: Struggling to gather information*

Establishing the exact details of the decision in question was challenging. This included establishing what information needed to be provided to an assessee about the decision, such as what the available options were for them to choose between:

*“When it is difficult to ascertain just what the salient points (e.g. what are the risks around the choice options) which are necessary to know when making the decision*.” Occupational therapist

*“Some areas are so complex, e.g. clients who have businesses with financial arrangements that I am not familiar with.”* Clinical Psychologist

This information was sometimes gathered from other professionals:

*“The challenges when being asked to assess capacity when it is not clear what options are on the table (e.g. social workers wanting to know if someone has capacity to make decisions regarding their discharge but we do not know what the options available to the patient are so cannot actually fully assess).”* Clinical Psychologist

Gathering collateral information from family or friends was experienced as hard, including learning more about the person. Some participants had difficulty choosing the best wording for asking questions during assessment.

*Theme 4: Engagement and influence*

Challenges were noted in building and maintaining effective relationships between people involved in assessment, be this with the person being assessed, people in their social environment and with navigating coercion and undue influence from these third parties.

*“Enabling an environment where patients can be open (i.e. stopping family members/carers from influencing patient's responses)”* Speech and Language Therapist

*“Also, family members wanting to be present during the assessment can present difficulties. While this can put the person at ease and sometimes assist in the process, it is difficult to know how much the person is being "led" to provide certain answers by the family member.”* Social worker

*“If family members are present it can be difficult encouraging them to allow the person to answer for themselves / not correcting the person if they "get the answer wrong" / to prevent the individual from looking to their family for the answers”* Social worker

*Theme 5: Supporting the person during the assessment*

This theme included challenges in arranging a broad spectrum of types of support, which a person might need to maximise the performance when having their capacity assessed. Support for communication was the most frequently discussed difficulty.

*“Ensuring the most appropriate alternative/augmentative communication devices are in place and are loaded with appropriate language/ information about the various decisions to be made.”* Nurse

Anxiety during the assessment was seen as requiring special provisions, to allow a quality assessment:

*“…issues when people are anxious about what is about to happen. I find clients perform better when they are relaxed about the process and depends on how they respond to me”*

Nurse

This was compounded in more complex situations, such as at the end of life:

*“It can be difficult with some patients, particularly those with diagnoses of terminal illness, to establish whether an individual does not wish to discuss the future and their treatment or whether they lack capacity to make decisions on the issue.”* Doctor: healthcare of the elderly

*Theme 6: Diagnosis as imperative*

Diagnoses were important in a number of ways. Non-medical assessors of capacity wanted a diagnosis to have been made by the medical team involved and they wanted to know what that diagnosis was.

*“If no formal diagnoses”* Social worker

*“Assessing people that have obvious cognitive impairment but have never received a diagnosis”* Social worker

Others cited particular groups as needing special considerations during assessment (in particular intellectual disability, dementia, alcohol use disorders and executive function problems)…

*“lack of specialist knowledge ie. acquired brain injury”* Social worker

… and with more severe symptoms making assessment more complicated for some respondents.

*Theme 7: Navigating the functional test of capacity*

The assessment of functional decision-making inability (as part of the MCA 2005 section 3 test) was seen as generally challenging, with the using-and-weighing of information seen as particularly problematic:

*“The test is, inherently, entirely subjective when it comes to the 'weighing up' criteria in particular, which is the element of the test most relevant to most patients with psychiatric disorders.”* Psychiatrist

Demonstrating that the functional inability was caused by any impairment of brain or mind (known as the ‘causative nexus’) was difficult.

*“Causative nexus. Stipulating that it is as a result of the disorder”* Nurse

Decisions which appeared unwise presented problems, and it was difficult to be sure if mental capacity in the office setting translated to decisions taken in real life situations (sometimes called the “frontal lobe paradox” in the context of brain injury):

*“For more complex assessments it is often a challenge when P seems to be able to understand, retain, use/weigh information and communicate a decision but they have compromised executive functioning and will do completely the opposite of what they stated they will do.”* Social worker

*“seeing the whole picture, and not simply person's presentation on a given day, in a structured environment, where they are not challenged to make decision in a 'live/hot', real life situation.”* Occupational therapist

This extended to concerns about impulsivity:

*“assessing people with brain injury who have frontal lobe damage and may seem to be able to hold and make decisions, but who have impulsive behaviour which really makes one question if that is full capacity”* Occupational therapist

*Theme 8: Complicated presentations and pressing decisions*

A range of features of cases were cited as producing particularly difficult assessments. Factors about the decision itself made some assessments more pressurised, including when the health status of the person required the assessment to be completed quickly, when the person was experiencing a mental health crisis, or when the decision involved life sustaining treatment.

Setting the threshold of functional ability at which the person (P) would be viewed as capacitous was challenging, as was capacity judged as fluctuant or borderline:

*“on the cusp of capacity but they’re frightened to admit the gaps in their memory and understanding”* Social worker

*Theme 9: Legal knowledge and opinions*

Keeping up to date with relevant law was experienced as difficult and more training was sought, and performing capacity assessment for court cases was experienced as difficult. There were also criticisms of the MCA 2005 itself and queries about the true value of current practices of capacity assessment:

*“Real world decision making does not follow the legal structure i.e. the science about human’s decision making and resulting behaviour does not support the mca test well. The Act does not define what a decision is anywhere which makes some assessments hard to do authentically.”* Clinical psychologist

*I think that most decisions that people make are done without considering the salient points or logically weighing them up. I think that it is normal that people decide based on gut feelings rather than reason.”* Occupational therapist

*“a very inexact and subjective science, even if one knows the Mental Capacity Act inside out”.* Psychiatrist

## Discussion

This large-scale survey provides quantitative and qualitative insights into experiences of capacity assessment by members of different professions. To our knowledge, this is the largest survey of its kind in England and Wales and the first to explore the experiences of clinical psychologists and solicitors. We found differences between the professional groups for each of our main topics of interest: how often they assessed capacity, how competent they felt in assessing capacity, and how often they considered undue influence. Overall, the experiences of non-psychiatrist doctors and clinical psychologists seemed to have been the most divergent.

We found that non-psychiatrist doctors and clinical psychologists were less confident than other professionals in assessing capacity. Only 6.9% (2/30) of non-psychiatrist doctors rated themselves as assessing capacity ‘very well’, which was similar to the 4% (3/83) who rated themselves as ‘extremely competent’ within a previous vignette study of non-psychiatrist doctors ( Penn *et al.*, 2020). The finding for clinical psychologists is novel. Both findings may reflect the observation that non-psychiatrist doctors and clinical psychologists in our sample undertook fewer capacity assessments, relative to other professional groups. They may also reflect a relative lack of knowledge and training around the MCA 2005 ( Penn *et al.*, 2020; Schofield, 2008). This would be a concern if non-psychiatrist doctors and clinical psychologists felt less able to participate in the assessment process, given that our sample desired better access to psychological or speech and language therapist expertise, particularly for complex cases and more acknowledgement that the MCA is relevant to non-psychiatric decision making.

The responses also shed light on the notion of undue influence on capacity. While this has received scant attention within the empirical literature, professionals were most likely to report that they were ‘sometimes’ (56.9%; 347/610) or ‘quite frequently’ (18.0%; 110/610) concerned about undue influence. Solicitors were the most frequently concerned, which is perhaps unsurprising given that undue influence is most extensively scrutinised within legal cases. Psychiatrists and non-psychiatric doctors were least concerned, which may reflect less professional familiarity with the concept.

When we asked professionals who they thought were most vulnerable to undue influence, their responses were considerably varied. Older adults and people with learning disabilities, dementia and severe mental illness were the most frequently mentioned, but many emphasised that potentially anyone could be affected. Both findings suggest that undue influence is poorly researched, relative to its prevalence. This raises important concerns of potentially unmet needs which are important to decisional autonomy. Furthermore, a lack of guidance beyond the MCA code of practice was reported as a major challenge within our sample. Future research should therefore build on frameworks that can enable professionals to identify and support people who may be unduly influenced ( Craigie, 2020; Kong & Keene, 2018) using data from both court and non-court settings. Literature on relational autonomy may also provide useful background ( Ho, 2008; Series, 2015). 

Use of some kind of structured or psychological tools was made by around a third of respondents with most being proformas. It is likely that many of the template assessment documents produced by employers and other organisations are little more than itemised proformas listing principles of the MCA 2005 and the assessment criteria, rather than providing specific assessment strategies.

A small number of respondents used some tools designed to measure decisional abilities themselves, with the challenge of matching the content of the tool to the actual decisions being faced by P. The relative absence of use of other capacity-specific measures suggests that professionals or organisations do not see them as useful in clinical settings – unpacking the reasons for this would allow barriers to be addressed when producing future tools.

Respondents used standard psychological tests, mainly use full cognitive testing, cognitive screening tests or tests of executive function. The most common use of the MoCA might reflect cognitive assessment practices in health services, or might have alternatively been chosen specifically for questions of mental capacity, for reasons such as its usefulness in providing a snapshot of executive function. Recent work ( McWilliams *et al.*, 2020) has found that the MMSE and “IQ” were the most commonly discussed measures in healthcare professional evidence in the Court of Protection when determining capacity (though not the ACE). It is difficult to say whether selection of the measurement by professionals is most closely related to considerations of their properties to measure decisional function, impairment of brain or mind, or whether these tests are simply available and easy to administer. There is an opportunity to develop panels of tests of function, tailored to the needs of different patient groups, decisional types and professional groups.

Our qualitative analysis of free text responses highlighted a wide range of challenging situations which professionals can experience. Many participants described careful consideration of their own practice and the practical and organisational barriers they faced, such as finding adequate time to carry out meaningful assessment. Capacity assessment was seen as sometimes complex, requiring training and access to resources. Tailoring assessment to different presentations was challenging, as was being certain about the questions to ask and how to assess responses – especially concerning assessment of using-and-weighing information. Despite this, the levels of self-rated competence have weighted means that are fair.

Differences in experiences of capacity assessment between professional groups and settings emerged from the qualitative data, though the nature of our analysis was not designed to make formal comparisons. Examples included tussles over which professional held responsibility for a decision and how to resolve disagreement.

The main strength of the present study is the relatively large cross-sectional sample, with reasonable power to detect quantitative differences between the professional groups. Many of these professional groups (solicitors, clinical psychologists, social workers and nurses) have not been represented in a similar survey on mental capacity in England and Wales. Another strength is that we designed our free text questions to provide further context to the main three questions, which improved interpretability. We also had minimal missing data for the three main questions. Finally, approximately one in four survey respondents consented to be contacted about participation in follow-up interviews, to explore the themes in even more depth.

### Limitations

We also note some important limitations. First, although most professional groups were reasonably well represented, our total sample is overrepresented by social work professionals (302/562) and underrepresented by solicitors (8/562). Uneven sample sizes are not uncommon in observational studies and the standard errors did not vary much between groups, suggesting that the analyses were reasonably valid. To avoid misinterpretations, we have reported either averages and frequencies for specific professional groups, or weighted averages within the results section. Any unweighted summary statistics should be interpreted with caution.

Secondly, as we recruited partly through social media and could not obtain national mailing lists, it would have been impossible to estimate the number of eligible people who did not respond. Therefore, we cannot rule out selection bias and our results may not be an accurate representation of each profession. It is likely that professionals who were most engaged with the issues around assessment of mental capacity will have responded. To mitigate against this risk, the survey was designed to be short – and therefore attractive to other professionals. We also deliberately targeted professionals in generic and non-specialist workplaces. It is important to note that the challenges described in our themes will not have been felt by all respondents across the sample, and so cannot be regarded as majority concerns.

## Conclusions

In this study, we have explored experiences of capacity assessments within a large and diverse sample of professionals. Professionals were generally confident in their own abilities and this was positively associated with how often they conducted assessments. However, the assessment process can include a wide range of challenges. Our findings suggest that clinical psychologists and non-psychiatrist doctors could particularly benefit from support to conduct capacity assessments. Structured tools or psychological tests do not find a high level of use and their use appeared inconsistent. This may reflect the limits of tests and the contextual and interpretative nature of capacity assessment. Guidelines on the use of psychological tests would be valuable to clarify limits and improve consistency of use. Finally, undue influence was a common concern amongst all of the groups. To mitigate against concerns of unmet need, this will need to be explored further in future research and policy.

## Data availability

### Underlying data

We have restricted free text responses, due to concerns that this additional information could have made participants identifiable on the basis of their employment. We have been advised by the King’s College London Research Ethics Committee (REC) that this is consistent with institutional policy. Restricted data is available on reasonable request to the corresponding author ( kevin.ariyo@kcl.ac.uk). This is likely to be a request from researchers for the purposes of further research, upon submission of both a detailed study protocol and signing a data access agreement with our research team.

Figshare: Capacity Survey Anonymised Data and Questionnaire. https://doi.org/10.6084/m9.figshare.14697006.v1 ( Ariyo *et al.*, 2021).

This project contains the following underlying data:

- Capacity Survey Full Data Wellcome Version.xlsx

### Extended data

Figshare: Capacity Survey Anonymised Data and Questionnaire. https://doi.org/10.6084/m9.figshare.14697006.v1 ( Ariyo *et al.*, 2021).

This project contains the following underlying data:

- Professional perspectives final survey 070319.pdf

Data are available under the terms of the Creative Commons Zero "No rights reserved" data waiver (CC0 1.0 Public domain dedication).

## References

[ref-1] Ariyo K McWilliams A David A : Capacity Survey Anonymised Data and Questionnaire. *figshare.* Dataset.2021. 10.6084/m9.figshare.14697006.v1

[ref-2] Bennett DA : How can I deal with missing data in my study? *Aust N Z J Public Health.* 2001;25(5):464–469. 10.1111/j.1467-842X.2001.tb00294.x 11688629

[ref-3] Braun V Clarke V : Using thematic analysis in psychology. *Qual Res Psychol.* 2006;3(2):77–101. 10.1191/1478088706qp063oa

[ref-4] Braun V Clarke V : What can “thematic analysis” offer health and wellbeing researchers? *Int J Qual Stud Health Well-being.* 2014;9:26152. 10.3402/qhw.v9.26152 25326092 PMC4201665

[ref-5] Brown RA Barber P Martin D : The Mental Capacity Act 2005: A Guide for Practice.Learning Matters,2015. Reference Source

[ref-6] Clarke V Braun V : Teaching thematic analysis: Overcoming challenges and developing strategies for effective learning. *psychologist.* 2013;26(2):120–123. Reference Source

[ref-7] Cohen J : Eta-squared and partial eta-squared in fixed factor ANOVA designs. *Educ Psychol Meas.* 1973;33(1):107–112. 10.1177/001316447303300111

[ref-8] Craigie J : Undue Influence in decision-making support for people with mental disabilities: A scoping paper.In Press,2020. 10.1093/medlaw/fwaa041 PMC8351318 34160030

[ref-10] Folstein MF Folstein SE McHugh PR : “Mini-mental state”. a practical method for grading the cognitive state of patients for the clinician. *J Psychiatr Res.* 1975;12(3):189–198. 10.1016/0022-3956(75)90026-6 1202204

[ref-12] Ho A : Relational autonomy or undue pressure? Family’s role in medical decision‐making. *Scand J Caring Sci.* 2008;22(1):128–135. 10.1111/j.1471-6712.2007.00561.x 18269432

[ref-13] Jayes M Palmer R Enderby P : Evaluation of the MCAST, a multidisciplinary toolkit to improve mental capacity assessment. *Disabil Rehabil.* 2020a;1–8. 10.1080/09638288.2020.1765030 32449375

[ref-14] Jayes M Palmer R Enderby P : How do health and social care professionals in England and Wales assess mental capacity? A literature review. *Disabil Rehabil.* 2020b;42(19):2797–2808. 10.1080/09638288.2019.1572793 30739505

[ref-15] Keene AR Kane NB Kim SYH : Taking capacity seriously? Ten years of mental capacity disputes before England's Court of Protection. *Int J Law Psychiatry.* 2019;62:56–76. 10.1016/j.ijlp.2018.11.005 30616855 PMC6338675

[ref-16] Kong C Keene AR : Overcoming challenges in the Mental Capacity Act 2005: Practical guidance for working with complex issues.Jessica Kingsley Publishers,2018. Reference Source

[ref-17] Mandelstam M : Safeguarding adults and the law.Jessica Kingsley Publishers,2013. Reference Source

[ref-18] McCormick M Bose A Marinis T : Decision-making capacity in aphasia: SLT’s contribution in England. *Aphasiology.* 2017;31(11):1344–1358. 10.1080/02687038.2017.1355441

[ref-19] McWilliams A Fleming SM David AS : The Use of Neuroscience and Psychological Measurement in England's Court of Protection. *Front Psychiatry.* 2020;11:570709. 10.3389/fpsyt.2020.570709 33364988 PMC7750429

[ref-20] Mioshi E Dawson K Mitchell J : The Addenbrooke's Cognitive Examination Revised (ACE‐R): a brief cognitive test battery for dementia screening. *Int J Geriatr Psychiatry.* 2006;21(11):1078–1085. 10.1002/gps.1610 16977673

[ref-21] Nasreddine ZS Phillips NA Bédirian V : The Montreal Cognitive Assessment, MoCA: a brief screening tool for mild cognitive impairment. *J Am Geriatr Soc.* 2005;53(4):695–699. 10.1111/j.1532-5415.2005.53221.x 15817019

[ref-22] National Institute for Health and Care Excellence: Decision-making and mental capacity.2018. Reference Source

[ref-23] Norman A Moore S Wotus R : Supported Decision Making: Brain injury case managers' experience of mental capacity and the mental capacity act.Plymouth, UK: Plymouth University.2018. 10.13140/RG.2.2.35575.73128

[ref-24] Penn D Lanceley A Petrie A : Mental capacity assessment: a descriptive, cross-sectional study of what doctors think, know and do. *J Med Ethics.* 2020; medethics-2019-105819. 10.1136/medethics-2019-105819 32647042

[ref-25] Schafer JL : Multiple imputation: a primer. *Stat Methods Med Res.* 1999;8(1):3–15. 10.1177/096228029900800102 10347857

[ref-26] Schofield C : Mental Capacity Act 2005 -- what do doctors know? 2008;48(2):113–116. 10.1258/rsmmsl.48.2.113 18533570

[ref-27] Series L : Relationships, autonomy and legal capacity: Mental capacity and support paradigms. *Int J Law Psychiatry.* 2015;40:80–91. 10.1016/j.ijlp.2015.04.010 25982964

[ref-29] Wechsler D : Manual for the Wechsler adult intelligence scale.1955. Reference Source

