# Supplementary material for: Experiences of assessing mental capacity in England and Wales: A large-scale survey of professionals
Source: Wellcome Open Res. 2021 Jun 10;6:144. [Version 1] doi: 10.12688/wellcomeopenres.16823.1 (PMC8246303; doi:10.12688/wellcomeopenres.16823.1)
Supplement: Peer review history

# Peer review: Reviewer response for version 1

**DOI:** 10.21956/wellcomeopenres.18558.r44466

**Published:** 2021-06-30

**Authors:** Peter Bartlett

**Affiliations:** 1 School of Law and Institute of Mental Health, University of Nottingham, Nottingham, UK

This reports the findings of a questionnaire on professionals’ experiences of assessing mental capacity in England and Wales. The questionnaire is short, and largely quantitative (with some short spaces to add in qualitative comments).

 The paper points up the strengths and weaknesses of the methodology selected. On the plus side, there is a high number of responses – n=611 – allowing for a sensibly wide array of responses. That no doubt flows in part from the fact the questionnaire is short and to the point. That in turn, however, means that there is not much by way of nuance in the findings. It is interesting that significant numbers of assessors are concerned about coercion from others; one would sensibly wonder, however, whether this was consistent across types of decisions for which assessments were being made. Was there more concern about undue influence in financial decisions or consent to treatment decisions? A significant number of professionals used structured instruments. Did they always do so, or only sometimes? Did that depend on the sort of client they were assessing, or on the type of decision, or what? Does the confidence of the assessor in their ability depend on the sort of judgment (or sort of client) that is being assessed? The structure of the survey does not allow this to be explored. One of the advantages of a big number of responses is that sort of analysis should be statistically viable. The structure of the questionnaire means that this sort of analysis is not possible here. 

 Similarly, because of the questionnaire format, it has not been possible for the researchers to probe into the qualitative statements. There are the beginnings of some quite interesting themes that are identified, but I confess I am left wanting more detail and exploration from the responders. 

 To be clear – the researchers never seem to make claims on these matters that are not supported by their findings. The frustration is that the structure chosen is limiting – to which the researchers might well respond that a more detailed structure would have reduced the response rate, creating a different set of limitations on the data.

 Even as it stands, there are questions about selection. There would seem to be a good number of social workers, psychiatrists, nurses and clinical psychologists, but by the time we get to speech therapists (12) and solicitors (8), it is fair to wonder what deductions can safely be drawn regarding these groups. Even for the larger groups, some additional discussion of recruitment would be helpful. I appreciate that with this sort of ‘cattle call’ survey (ie., whoever pitches up can fill it in) it is difficult to say much about the representativeness of the sample (and the authors acknowledge that selection bias may be an issue), but some more explanation of recruitment would not go amiss.

Is the work clearly and accurately presented and does it cite the current literature?

Yes

If applicable, is the statistical analysis and its interpretation appropriate?

Partly

Are all the source data underlying the results available to ensure full reproducibility?

Yes

Is the study design appropriate and is the work technically sound?

Yes

Are the conclusions drawn adequately supported by the results?

Yes

Are sufficient details of methods and analysis provided to allow replication by others?

Yes

Reviewer Expertise:

Mental health/mental capacity law; human rights.

I confirm that I have read this submission and believe that I have an appropriate level of expertise to confirm that it is of an acceptable scientific standard.

# Peer review: Reviewer response for version 1

**DOI:** 10.21956/wellcomeopenres.18558.r44444

**Published:** 2021-06-29

**Authors:** John Dawson

**Affiliations:** 1 Faculty of Law, University of Otago, Dunedin, New Zealand

This study reports the results of a survey of a large sample of UK health professionals concerning their experiences of capacity assessment conducted under the MCA 2005. The study is soundly-designed, properly-analysed and well-reported. It has clear aims. The methods are well-described, including the statistical analysis (although I am not qualified to assess the quality of that analysis). The study's limitations, including possible selection bias, are fairly discussed.

 Particular strengths include the large sample (600+ respondents), drawn from several health disciplines, with sufficient numbers for their responses to be usefully compared; plus the inclusion of many respondents who conduct a large number of capacity assessments (25+ a year). This means that, between them, the respondents have a lot of experience in this field.

 The study is particularly useful as a composite portrait of the current challenges facing UK health professionals involved in capacity assessment, especially the practical challenges. In a rather 'gritty' manner, it brings the process to life. It reaches important findings concerning: the frequency of concerns about undue influence; the relative infrequency of assessors being required to testify about their findings in court; the infrequent use of structured capacity assessment tools; and the distinct experiences of non-psychiatrist doctors and psychologists.

 Overall, the study usefully complements the large body of more theoretical writing on the capacity assessment process and on the law established by the MCA. It should be useful in teaching and as reading material in professional courses.

 Open access is provided to the study's quantitative data, while the qualitative data can be made available in appropriate cases, on request. Generally, the study provides a good example of the value of open access publishing and data sharing.

 I have no specific changes to recommend.

Is the work clearly and accurately presented and does it cite the current literature?

Yes

If applicable, is the statistical analysis and its interpretation appropriate?

I cannot comment. A qualified statistician is required.

Are all the source data underlying the results available to ensure full reproducibility?

Partly

Is the study design appropriate and is the work technically sound?

Yes

Are the conclusions drawn adequately supported by the results?

Yes

Are sufficient details of methods and analysis provided to allow replication by others?

Yes

Reviewer Expertise:

Mental health law; mental capacity law; socio-legal research methods and ethics.

I confirm that I have read this submission and believe that I have an appropriate level of expertise to confirm that it is of an acceptable scientific standard.
